# Steller sea lion (*Eumetopias jubatus*) consumption of ocean age-0 Chinook salmon (*Oncorhynchus tshawytscha*) along the northwest coast of Washington State

**DOI:** 10.1371/journal.pone.0334612

**Published:** 2025-11-12

**Authors:** Zoë K. Lewis, Benjamin W. Nelson, Adrianne M. Akmajian, Jonathan J. Scordino, Elizabeth M. Allyn, Sarah Brown, Dietmar Schwarz, Alejandro Acevedo-Gutiérrez

**Affiliations:** 1 Biology Department, Western Washington University, Bellingham, Washington, United States of America; 2 Independent Consultant, Seattle, Washington, United States of America; 3 Makah Fisheries Management, Makah Tribe, Neah Bay, Washington, United States of America; 4 Washington Department of Fish and Wildlife, Olympia, Washington, United States of America; University of Nevada, Reno, UNITED STATES OF AMERICA

## Abstract

The decline of wild Chinook salmon (*Oncorhynchus tshawytscha*) in the Pacific Northwest is concerning due to their critical role in the culture, economy, and ecology of the region, and the endangered species status of some of the evolutionarily significant units. Decline in Chinook stocks has been partially attributed to increases in pinniped abundance. The northwest coast of Washington State, USA, provides year-round habitat to early marine-phase Chinook salmon from multiple stocks and habitat for increasingly abundant Steller sea lions (*Eumetopias jubatus*). We estimated the Chinook salmon biomass consumed by Steller sea lions along the northwest coast of Washington using diet data obtained via DNA metabarcoding from scat and a prey consumption model. Between December 2020 and August 2021, Steller sea lions consumed 284 metric tons (95% PI: 191–417 t) of Chinook salmon. A set of experimental models were used to estimate the consumption of age-0 Chinook salmon, and the base model estimated 146 t (95% PI: 93–221 t) of ocean age-0 Chinook—or approximately 2,064,418 (95% PI: 1,431,524–2,932,922) individual ocean age-0 Chinook— were consumed during the study period. While precise consumption values should be interpreted with caution due to high uncertainty highlighted by sensitivity analyses, our results suggest that Steller sea lions contribute to the low marine survival rates of early marine-phase Chinook salmon at a higher rate than previously estimated. The high uncertainty in model estimates, compounded by assumptions and limitations arising from data gaps, highlights the need for further research on both predator and prey populations in the region.

## Introduction

Survival and productivity of Chinook salmon (*Oncorhynchus tshawytscha*) populations along the U.S. West Coast have declined precipitously despite large reductions in commercial, recreational, and tribal fisheries [1–3]. Increased natural mortality has thus been cited as a cause of Chinook salmon decline [4–7]. Specifically, natural mortality of ocean-age-0 salmon (defined as those that have spent less than a year in the ocean), has been implicated as a driver in decreased abundance of both wild- and hatchery-origin Chinook salmon [4,8–11]. Coded wire tag recovery of hatchery Chinook salmon has confirmed decreases in juvenile survivability along the Washington coast [12], a region used by a diverse mixture of Chinook stocks during their early marine phase [13–15]. Natural mortality at this life stage is due to a combination of factors that vary by location, such as ocean temperatures, salinity, weather conditions, prey availability, and predation [6,9–11,16–23]. Hypothesis-driven statistical models of Chinook marine survival indicate that predation, hatchery release timing and anthropogenic impacts (such as habitat degradation) may be the strongest predictors of decline [6]. Further, the effects of climate change on early marine phase salmon through a combination of bottom-up and top-down trophic processes, including marine mammal predation, may limit the population growth of the species [10]. Therefore, determining causal mechanisms of early mortality is critical for conservation and recovery efforts, and sustainable harvest management.

In the Pacific Northwest, three pinniped species, harbor seals (*Phoca vitulina*), California sea lions (*Zalophus californianus*) and Steller sea lions (*Eumetopias jubatus*), consume Chinook salmon at varying rates [24–29]. In the Salish Sea, harbor seal abundance has been negatively correlated to Chinook salmon survival [6]. Consequently, harbor seals have been the focus of many studies quantifying consumption of Chinook salmon in the region and along the coast of the Eastern North Pacific Ocean [24,26–28,30]. However, the effects of predation on Chinook salmon by other pinniped species, like the Steller sea lion (*Eumetopias jubatus*), have received less attention [31] or have been seen as relatively insignificant in comparison to harbor seal impacts [24,27,28]. Chinook salmon from populations originating in the Puget Sound and the Columbia River listed under the Endangered Species Act (ESA) migrate through or reside in the productive marine waters off the northwest Washington State coast year-round [2,5,15,16,32–34]. In the same region, Steller sea lion abundance increased at a rate of nearly 8% per year between 2010–2017 [35]. Steller sea lions in Washington are part of the Eastern distinct population segment (EDPS), which was delisted from the ESA in 2013 following a population growth of 4.2% per year between 1979–2010 [36,37]. Given the increasing evidence that thriving pinniped populations may be impacting salmon populations [6,11,20,25,38,39], the temporal association between increasing Steller sea lion abundance and decreased Chinook salmon survival in the region deserves further study.

Quantifying predation depends on the demographics, abundance, and spatial overlap between predator and prey species, which is influenced by both abiotic and biotic factors [40,41]. Biomass models are useful tools to estimate the perceived impact of predation and must be regionally specific and updated to reflect ecosystem changes [28,42–44] and to incorporate updated diet detection and analysis methods as available [29,30,45]. Previous biomass modeling research using diet data collected along the coast of northwest Washington during 2010–2013 [46] suggests that consumption of Chinook salmon by Steller sea lions was minimal relative to other marine mammal species [11,28]. This initial biomass study utilized hard prey remains from scats that were identified to the Salmonid family [46] and extrapolated to estimate the species-level proportion of salmon in Steller sea lion diet. Subsequent molecular analysis of the same salmon bones suggests that Chinook specific predation may be higher than previously thought [31]. Recent advancements in diet reconstruction methods using DNA metabarcoding has allowed for greater species-level identification of prey items, including salmon, which has vastly improved diet estimation methods [25,29,45,47,48]. Despite biases in DNA metabarcoding studies [29,45,49–52], recent summaries of harbor seal metabarcoding data emphasize improved biomass estimate accuracy of DNA metabarcoding over hard parts diet analysis methods [45]. While previous studies implicating harbor seals as the main pinniped contributor to Chinook salmon predation used DNA metabarcoding as the quantitative diet reconstruction method, Steller sea lion diet data was extrapolated from hard remains [27,28] and thus is likely to be underestimated.

Due to shifts in ecological conditions and advancements in diet reconstruction and modeling methods over the last decade, our overall aim is to update estimates of Chinook salmon biomass consumed by Steller sea lions along the northwest coast of Washington and to quantify their consumption of ocean age-0 Chinook salmon to better understand the impacts of Steller sea lion predation in the region. Specifically, our objectives are: 1) to model the contribution of Chinook salmon (all age classes) to Steller sea lion diets on the northwest Washington coast from December 2020 through August 2021 using DNA metabarcoding; 2) to estimate the total biomass (in metric tons) of prey consumed, as well as the total Chinook biomass consumed; and 3) to model the contribution of ocean age-0 Chinook to Steller sea lion diet (by combining DNA metabarcoding with ages of salmon determined via hard parts analysis) and model the biomass, as well as the number of individual ocean age-0 Chinook salmon consumed during the study period.

## Materials and methods

### Study site and collection of Steller sea lion scat

**Fig 1:**
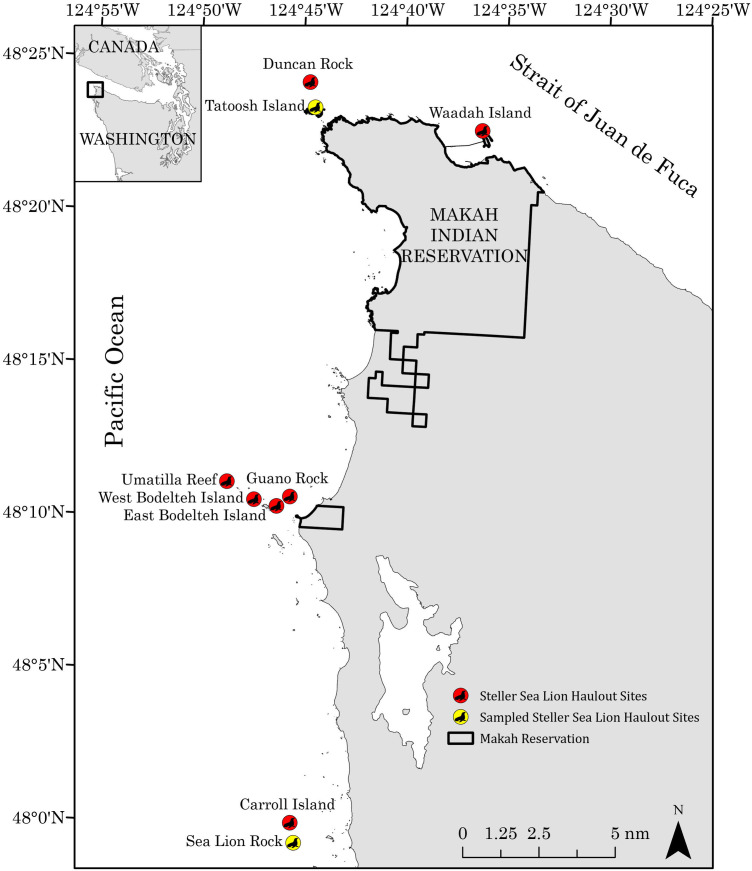
Study area and haulout site map. Haulout sites surveyed between December 2020–August 2021 to inform Steller sea lion abundance estimates along the northwest coast of Washington State. Sites where scat samples were collected are marked in yellow. Abundance estimates were modeled on days where haulout counts were conducted across all of the marked (both yellow and red) locations between 2020–2021. Source: Makah reservation boundary was downloaded from the **U.**S. Census Bureau American Indian/Alaska Native Areas/Hawaiian Home Lands shapefile (https://www.census.gov/geographies/mapping-files/time-series/geo/cartographic-boundary.html). Washington state boundary shapefile was originally sourced from the **U.**S. Census Bureau as well, but was modified by the Makah Tribe GIS Department. Canada boundary shapefile used in the inset was sourced from Statistics Canada (https://www12.statcan.gc.ca/census-recensement/2021/geo/sip-pis/boundary-limites/index2021-eng.cfm?year=21).

This study was conducted along the outer coast of northwest Washington State, at the confluence of the Strait of Juan de Fuca and the Pacific Ocean (Fig 1). This region hosts a rapidly growing population of Steller sea lions that utilize nine haulout sites throughout the year (Fig 1) [35,53]. Scat samples were collected from Tatoosh Island and Sea Lion Rock, because these sites have been previously sampled for Steller sea lion scats, have few California sea lions (*Zalophus californianus*) present, have observed overlap in branded individuals (suggesting frequent interchange of individuals across sites), and are the safest haulouts to land biologists for scat collection [31,54,55]. Scats were primarily collected from Tatoosh Island, with additional samples gathered from Sea Lion Rock (approximately 45 km south) when sea lions were absent or inaccessible at Tatoosh Island (S1 Table). Scat samples were collected under Marine Mammal Protection Act research permit #23970. To access the haulout sites, we used Special Use Permit #20008 for Sea Lion Rock in the Washington Maritime National Wildlife Refuge Complex and permission from the Makah Tribe for Tatoosh Island. Haulout counts were conducted via vessel-based surveys of all nine haulout sites (Fig 1) at least once monthly between March 2020 and August 2021. Scat collected at Tatoosh Island and Sea Lion Rock were assumed to be representative of Steller sea lions across all haulout sites (Fig 1), as Steller sea lions are multiple central-place foragers [56] that move between haulouts within the region.

Scat collection and processing followed methods described in Thomas et al. [25]. Fresh scats were scooped with wooden spoons into either 500 mL Histoplex containers lined with fine mesh (0.25 mm) nylon paint strainer bags or directly into sterile, plastic Whirl-pak bags. A unique wooden spoon was used for each scat sample and the collector changed dirtied gloves to prevent cross-scat genetic contamination. After collection, scats were frozen at −20˚ C within 6 hours to preserve DNA quality. Frozen scats were transferred into a paint strainer bag then manually homogenized in ethanol until thoroughly homogenized. DNA extraction from the scat-ethanol mixture was conducted with QIAGEN QIAamp DNA Stool Mini Kit using adjusted protocols for pinniped scat [57]. Paint strainer bags containing the hard remains were sealed and washed in a residential style washing machine on a gentle setting [58]. Remains were carefully removed from paint strainer bags, rinsed through nested sieves (2, 1, and 0.5 mm) to further remove fecal material and then placed in isopropyl alcohol [59]. Finally, all remains were dried, except for cephalopod remains (pens and beaks) which remained stored in isopropyl alcohol to preserve shape [59].

### Prey identification, diet reconstruction and salmon aging

DNA metabarcoding analysis was performed to estimate the proportion of DNA in the scat sample that originated from salmon species, known as the relative read abundance (RRA) [29]. Despite known biases in current metabarcoding used for quantitative purposes [60,61], we used RRA to estimate diet proportion to be consistent with other studies investigating marine mammal consumption of Chinook salmon [24,27,28,45,52,62]. Our selection of RRA was also driven by the ability for DNA metabarcoding to identify a higher number of prey items to species level [25,45,63]. Methods for using DNA metabarcoding for studying pinniped diets have been previously described in Thomas et al. [45,64] and modifications implemented for Steller sea lions along the northwest coast of Washington are described in Lewis [63]. Two PCR assays targeting the mitochondrial 16S rRNA (16S) and Cytochrome Oxidase I (COI) regions were performed on DNA extracted from the scats. Bioinformatics were performed to proportionally decontaminate samples and assign DNA sequences to species using a custom BLAST reference database with a 99% assignment level [63]. Salmon species RRA was assigned using the proportion of DNA assigned as Pacific salmon (*Oncorhynchus* spp., pooled for all species) by the 16S assay divided by the proportions of individual salmon species found via the COI assay [25].

Hard parts from scat samples were examined and all Pacific salmon prey remains were separated. All recovered Pacific salmon otoliths and vertebrae were graded and measured using an ocular micrometer to determine size of the salmon [65]. Otolith and vertebrae identification, along with comparison to reference specimens and examination of morphological differences, were used to determine the age class of Pacific salmon remains. These identified hard parts were then classified as either: 1) ocean age-0 Pacific salmon (using the European aging system [66]) defined as remains from fish determined to be < 300 mm [30] or 2) “other” aged Pacific salmon. This second category encompasses prey remains of Pacific salmon that were assigned as adult (>375 mm), that were too degraded to definitively assign age class or remains from fish estimated to be between 300 and 375 mm. It is important to note that with the high likelihood of degraded bones, an undetermined proportion of the “other” class could also be from age-0 remains, biasing the age-0 Pacific salmon proportion low.

### Steller sea lion total prey and Chinook biomass consumption estimation

We used Bayesian inference for beta regression with inflation at zero and one (Zero/One Inflated Beta Regression or ‘zoib’) [67] to quantify uncertainty and calculate confidence intervals around the estimated contribution of Chinook (all age classes) to Steller sea lion diet based on the RRA proportions. Detailed descriptions of the ‘zoib’ modeling methods can be found in supplementary information (S1 Appendix). The ‘zoib’ model produced 8,000 sample replicates, which were then bootstrapped using Monte Carlo simulations (n = 2,000 replicates per season, 6,000 replicates total) to determine 95% credible intervals (CI) of seasonal and annual consumption estimates.

To estimate the biomass in metric tons (t) of total prey consumed by Steller sea lions along the northern coast of Washington between December 2020–August 2021, we used a set of nested equations to build a biomass model to account for demographic and seasonal variations in both abundance and consumption. Seasonal prey consumption was calculated with the following equations, adapted from Scordino et al. [31].


$$\begin{array}{c} B_{x}=\;\sum_{z}\frac{w_{z}\times c_{z}\times d_{x}\times n_{x}\times p_{x,z}\times f_{x}}{\text{1}\text{0}\text{0}\text{0}} \end{array}$$ (1)

where; *B*_*x *_= seasonal biomass consumed,

*x* = season (winter, spring or summer),

*z* = the demographic group (adult male, adult female, juvenile male, and juvenile female),

*w*_*z* _= the estimated average body weight of sea lion in kilograms of demographic group *z*,

*c*_*z*_ = percentage of body weight that sea lion demographic group *z* eats per day,

*d*_*x*_ = the number of days in season *x*,

*n*_*x*_ = the average count of age-1 + sea lions hauled out in the survey area during season *x*,

*p*_*x,z* _= the proportion of sea lions counted in season *x* of demographic group; and

*f*_*x*_ = the correction factor for converting the haulout count to the total abundance of Steller sea lions in the environment of northwest Washington during season *x*.

Then, the seasonal biomass *B*_*x*_ was summed across winter, spring and summer to estimate the total biomass consumed, *B*_*a*_ during the study.


$$\begin{array}{c} B_{a}=\;\sum_{x}B_{x}\; \end{array}$$ (2)

The variables listed in equation 1 were modeled using independent functions for each variable as follows. Two conditional parameters used to represent the bioenergetic demands of individual Steller sea lions, *w*_*z*_, average body weight and *c*_*z*,_ percentage of body weight consumed, were previously determined in Winship et al. [68] and used in prey consumption modeling for the northwest coast of Washington [55]. The total count of Steller sea lions across all sites, n_x_, was modeled using a negative binomial distribution to obtain average Steller sea lion counts by season from counts conducted between 2020–2021 [55]. The correction factor *f*_*x*_ for the haulout count for sea lions in northwest Washington during season x was based on results in Olesiuk [69]. The seasonal correction factors were modeled using normal distributions based on the following proportions: 36% of individuals were counted during haulout surveys in winter, spring, and fall, and 67% were counted during surveys conducted in the summer. Seasonal demographics and juvenile sex proportions represented by variable *p*_*x,z*_ were modeled with beta distributions using previous demographic counts for the region [46] and published survival rates for juvenile Steller sea lions [55,70].

**Fig 2:**
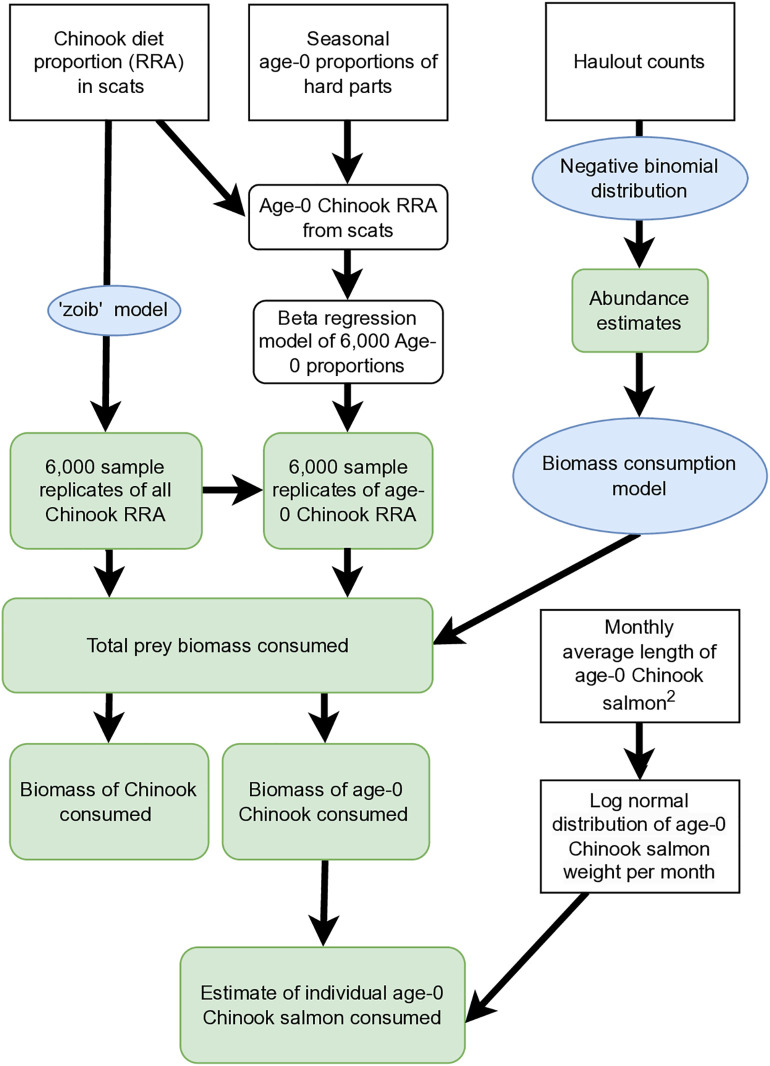
Modeling schematic. Modeling schematic detailing steps from scat collection to model outputs. Zero/one inflated beta regression with Bayesian inference was used to model sample replicates of Chinook salmon diet proportion (n = 2,000 per season) combined with prey consumption model (n = 2,000 replicates per season) to estimate biomass of Chinook consumed. White boxes show data inputs. Blue ovals represent modeling processes. Green boxes represent data generated from modeling.


To estimate the biomass consumption of Chinook salmon, Monte Carlo simulations were used to create 2,000 replicates of total biomass consumed per season (Eq. 1) and multiplied by the seasonal Chinook RRA proportion replicates (as determined by the ‘zoib’ model, Fig 2) to generate the average estimated biomass consumption of all Chinook and 95% predictive intervals (PI) (Eq. 3).

$$C_{x}=B_{x}\times r_{x}$$ (3)

where; *C*_*x*_* *= seasonal Chinook biomass consumed, *B*_*x*_ = seasonal biomass consumed; and *r*_*x*_ = seasonal relative read abundance (proportion) of Chinook consumed determined via relevant ‘zoib’ model.

Finally, seasonal biomass consumed, *C*_*x*_, was summed across all seasons to get the total biomass, *C*_*a*_, of Chinook salmon consumed by Steller sea lions during the study period.


$$\begin{array}{c} C_{a}=\;\sum_{x}C_{x} \end{array}$$ (4)

### Consumption of age-0 Chinook salmon

The proportion of age-0 Chinook salmon consumed by Steller sea lions along the coast of northwest Washington was calculated by dividing the number of age-0 remains by the total number of Pacific salmon remains recovered each season. These proportions formed the base model scenario. Due to assumptions and uncertainties surrounding the age-0 proportion estimates, additional models were run with adjustments of 10%, 25%, and 50% from the original proportions. A bootstrapping method was used as a sensitivity test to assess statistical differences between the base and alternative models. Due to the non-normality of sample replicates for all models, replicates were log transformed prior to bootstrapping analysis, and therefore the interpreted observed difference, is the inverse log transformed, and is reported as percent change from base model.

To estimate the age-0 Chinook RRA, seasonal age-0 proportions were modeled using beta regression to generate 2,000 sample replicates per season, parameterized by the sample size of recovered hard parts. These replicates were then multiplied by the Chinook RRA sample replicates (derived from the ‘zoib’ model) to obtain 2,000 age-0 Chinook RRA replicates per season. The biomass of age-0 Chinook salmon consumed was calculated similarly to the total Chinook biomass consumed, by multiplying sample replicates from the seasonal biomass model (Eq. 1) by age-0 Chinook RRA replicates (Eq. 3). Monte Carlo simulations were used to produce 6,000 sample replicates (2,000 per season) to generate the mean biomass consumption of age-0 Chinook estimate and 95% predictive intervals (PI) for the base and adjusted models.

To estimate the count of age-0 Chinook salmon individuals consumed, monthly age-0 Chinook consumption estimates were divided by estimated monthly age-0 Chinook weight. More specifically, the set of nested consumption functions (Eq. 5 and 6) divided by the monthly age-0 Chinook weight functions within a season (Eq. 7) to determine the seasonal distributions of individual Chinook salmon consumption (Eq. 8). Seasonal consumption of age-0 Pacific salmon was then summed across all months to get total consumption of individual Chinook salmon consumed across the study period (Eq. 9). Monte Carlo simulations were used to produce 6,000 sample replicates (2,000 per season) to generate the mean number of age-0 Chinook consumed and 95% predictive intervals (PI) for the base model.

Estimates of ocean age-0 Chinook lengths along the coast of Washington were taken directly from length estimates previously used in Chinook salmon consumption for Steller sea lion biomass modeling in the region by Chasco et al. [28]. Chasco et al. provide estimates of age-0 length based off growth rates and sizes [15] of Chinook salmon out migrating from the Salish Sea. Length distributions were modeled using a log normal distribution to account for variation in size, and transformed into individual salmon weight using the allometric model of length to mass relationships reported in Nelson et al. [30]. Following these parameters, the mass of ocean age-0 Chinook individuals ranged from 0.01–0.3 kg. Additional description of length and weight calculations, as well as monthly distributions can be found in S2 Appendix.

To directly compare to previous consumption rates of individual Chinook salmon consumed by Steller sea lions previously reported by Chasco et al. [27] we calculated the daily consumption of individual of age-0 Chinook salmon by each Steller sea lion demographic group monthly. Daily consumption estimates of age-0 Chinook were calculated by multiplying daily consumption rates by the age-0 sample proportion replicates, then dividing by the monthly weight function (Eq. 10). To estimate daily consumption for the different demographic groups of the sea lions, we considered the individual metabolic requirements of each demographic group (i.e., the percentage of body weight eaten per day by each demographic group [71]), while using the same sample replicates of RRA for age-0 Chinook for each demographic calculation. We report the maximum daily average consumed for each demographic group (adult male, adult female, juvenile male, and juvenile female).


$$\begin{array}{c} B_{i}=\;\sum_{z}\frac{w_{z}\times c_{z}\times n_{x}\times p_{x,z}\times f_{x}\times d_{i}}{\text{1}\text{0}\text{0}\text{0}} \end{array}$$ (5)

$$C_{i}=B_{i}\times r_{x(i)}$$ (6)

$$m_{i}=\frac{\text{9}.\text{6}\text{1}\times {\frac{l_{i}}{\text{1}\text{0}}}^{\text{3}.\text{0}\text{7}}}{\text{1},\text{0}\text{0}\text{0},\text{0}\text{0}\text{0}}$$ (7)

$$A_{i}{=\;C}_{i}/\;m_{i}$$ (8)

$$\begin{array}{c} A_{a}=\;\sum_{i}A_{i} \end{array}$$ (9)

$$A_{z,i}=\;\frac{w_{z}\times c_{z}\times n_{x}\times p_{x,z}\times f_{x}\times r_{x(i)}}{\text{1}\text{0}\text{0}\text{0}\;\times {\;m}_{i}}$$ (10)

where; *B*_*i *_= monthly biomass of prey consumed,

*r*_*x(i)*_ = seasonal relative read abundance (proportion) of age-0 Chinook consumed, for the season in which month *i* occurs,

*d*_*i*_* *= the number of days in month *i*,

*C*_*j *_= seasonal age-0 Chinook biomass consumed (kg),

*m*_*i*_ = mass of individual age-0 Chinook consumed (kg) in month *i*,

9.61 = the alpha constant from the allometric length weight relationship in Nelson et al. (2019),

3.07 = the beta constant from the allometric length weight relationship in Nelson et al. (2019),

*l*_*i*_ = monthly average fork length in mm of age-0 Chinook salmon,

*A*_*i* _= monthly consumption of individual age-0 Chinook by Steller sea lions,

*A*_*a* _= full study consumption of individual age-0 Chinook by Steller sea lions; and

*A*_*z,i* _= daily consumption of Chinook individuals consumed by Steller sea lion demographic group *i.*

## Results

### Presence and age of Chinook salmon in Steller sea lion diet

**Fig 3:**
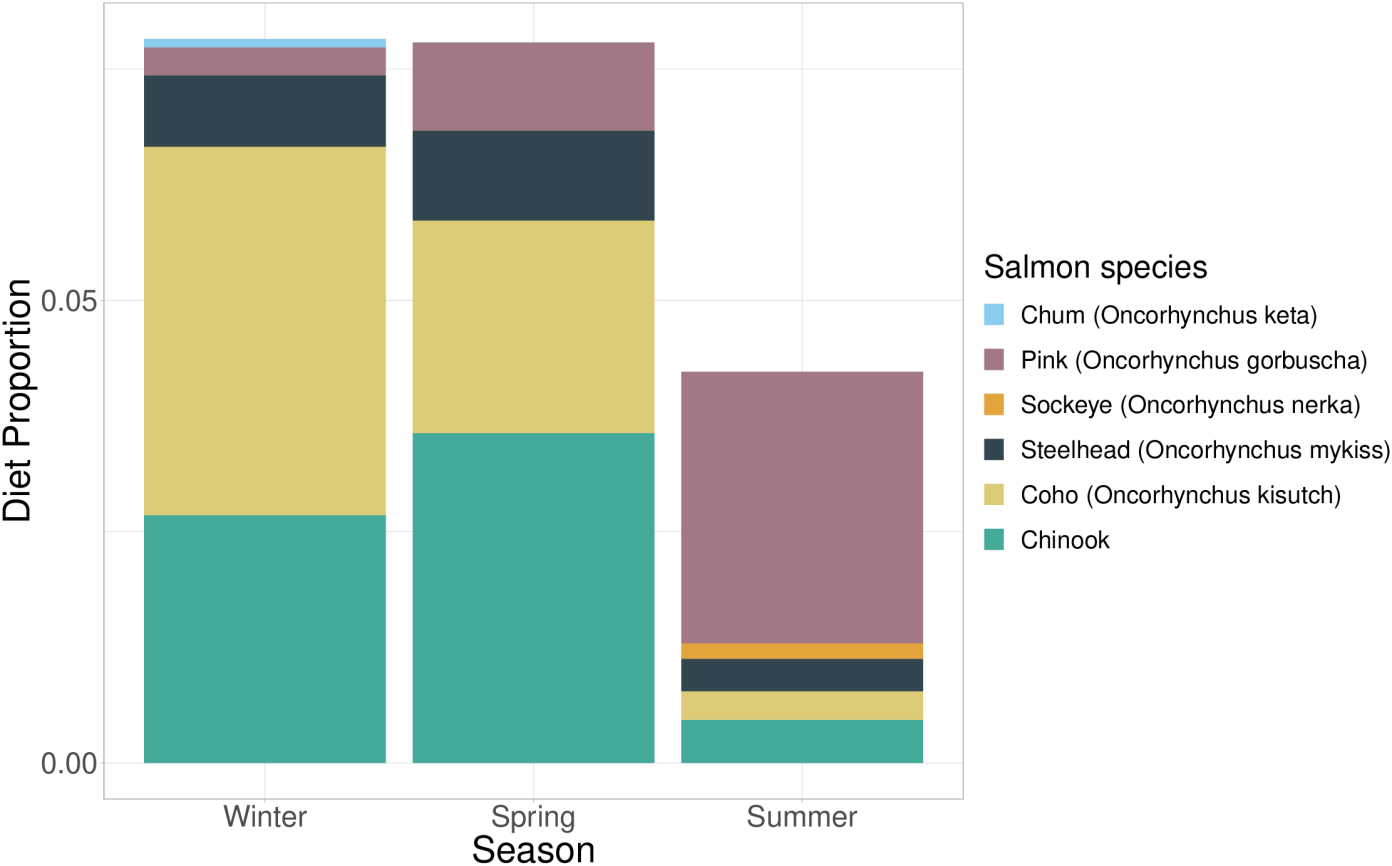
Relative read abundance of Pacific salmon in Steller sea lion diet. Bar plot of observed Pacific salmon diet proportions (RRA) from DNA metabarcoding analysis of Steller sea lion scats (n = 267) collected between December 2020–August 2021 in northwest Washington.

A total of 274 Steller sea lion scats were collected between December 2020–August 2021, with relatively even distribution (92 in the winter defined as December-February, 90 collected in the spring defined as March-May, and 85 in the summer defined as June-August). DNA metabarcoding of prey items was successful in 97.4% of samples (n = 267). Family-level salmon prey items were detected in 128 scats (47.9% FO, frequency of occurrence of Chinook DNA detected) via DNA metabarcoding analysis of the 16S rRNA. Of these, Chinook salmon DNA was identified in 71 (27% of total) collected scats. Chinook salmon were most prevalent in scats during spring and winter, and was highest relative to other salmon species in the spring (Fig 3). Chinook salmon DNA was detected at similar rates during in spring and winter, with 38.8% FO in the spring (35 of 90 scats) and 37% FO of scats in the winter containing Chinook DNA (34 of 92 scats). Summer detection of Chinook DNA was much lower, at 4.7% FO of scats (4 of 85 scats).

Pacific salmon hard parts were recovered from 62 of 274 scats (22%) with two of those scats containing salmon structures of disparate sizes, indicating multiple individual salmon were consumed. Therefore, a total of 64 salmon parts were used to determine age-0 proportions. A total of 33 hard parts were from age-0 individuals, leaving 31 hard parts of either unidentified age or adult age (Table 1). In other words, 51% of Pacific salmon remains were attributed to age-0 individuals. Age-0 Pacific salmon remain recovery was highest in the winter relative to other seasons (Table 1). Due to degradation, only 5 scats contained hard parts that could be identified to salmon species, with only one otolith identified to be from Chinook salmon. Using the otolith height regression equation determined by Nelson et al. [30] to calculate standard length, the estimated length of this individual Chinook was 423.8 mm.

**Table 1:** Seasonal proportions of age-0 Pacific salmon (*Oncorhynchus* spp.) hard parts.

Season	Pacific salmon hard remains recovered		Estimated Age-0 proportion (Base model inputs)	Proportions for sensitivity analysis					
	Total	Age-0		−10%	+10%	−25%	+25%	−50%	+50%
Winter	29	17	0.58	0.522	0.638	0.435	0.725	0.29	0.87
Spring	18	10	0.55	0.495	0.605	0.413	0.6875	0.275	0.825
Summer	17	6	0.35	0.315	0.385	0.263	0.438	0.175	0.525
**Overall**	**64**	**33**	**0.51**						

Seasonal proportions determined from age-0 hard parts recovered in Steller sea lion scats collected from the northwest coast of Washington State during this study in 2020–2021 (n = 64). Proportions for sensitivity analysis are the result of increasing or decreasing the estimated age-0 proportion by 10%, 25%, or 50%.

### Regional Steller sea lion abundance and total prey biomass consumed

**Fig 4:**
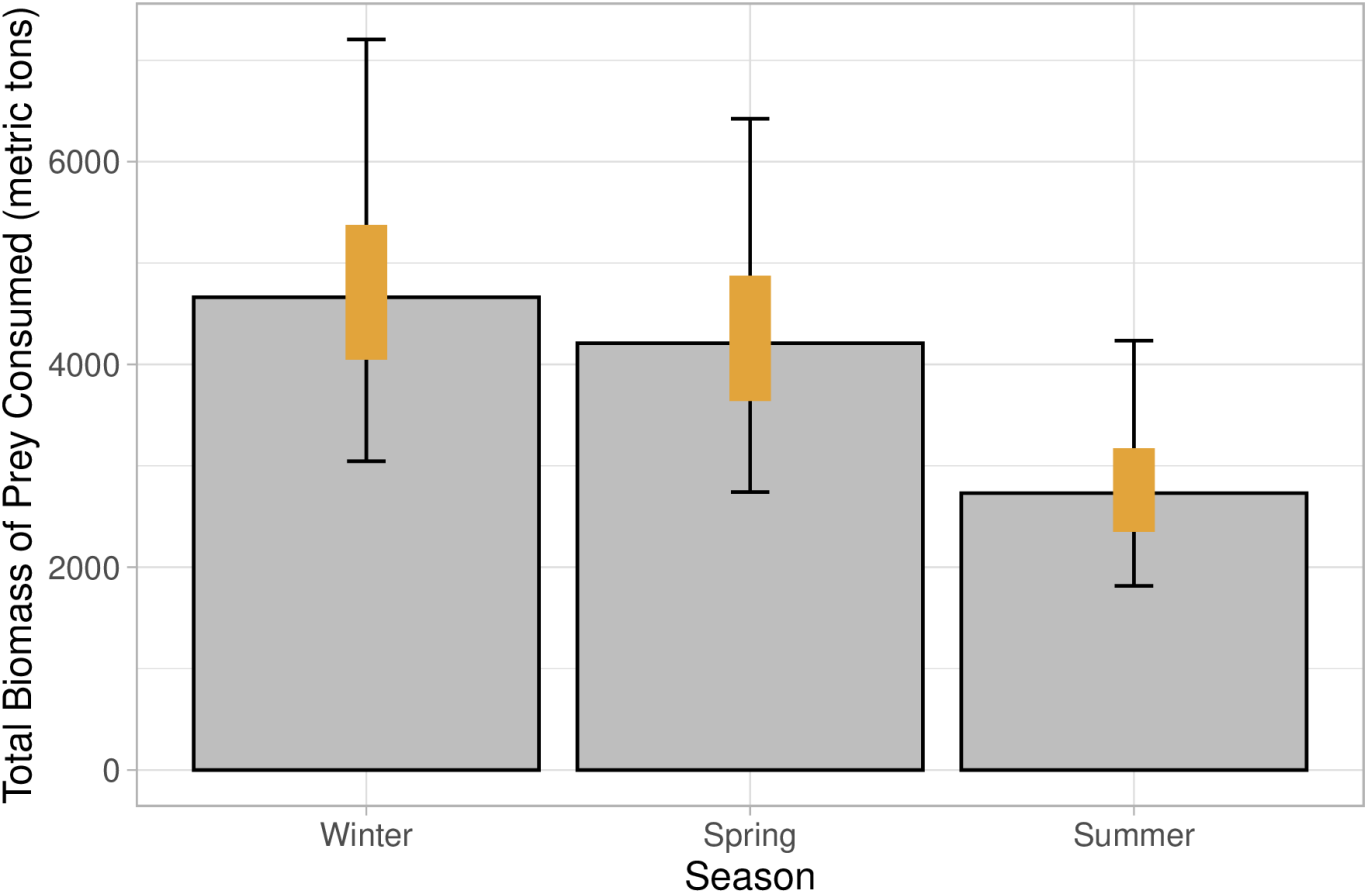
Metric tons of prey consumed by Steller sea lions along the northwest coast of Washington State from December 2020-August 2021. Bars represent model derived median, orange bars represent 50% confidence intervals, and black error bars represent 95% confidence intervals.

Mean count data was modeled using a negative binomial distribution to estimate abundance of Steller sea lions at haulouts in northwest Washington during the study period (Table 2). Correcting the counts using the proportion of the population hauled out from Olesiuk [69] resulted in much higher abundance estimates in the study region in winter and spring than during summer (Table 2); note that abundances are not reported in the table, as the binomial distribution was used as an input for the consumption estimates, rather than a single estimated abundance, to incorporate uncertainty. The prey biomass model estimated 11,802 metric tons (t; 95% CI: 9,228–15,005 t) of total prey were consumed by Steller sea lions between December 2020 and August 2021. The seasonal biomass consumed varied (Fig 4), with winter and spring showing higher overall consumption of prey compared to summer: winter 4,662 t (95% CI: 3,046–7,205 t), spring 4,210 t (95% CI: 2,741−6,424 t), and summer 2,731 t (95% CI: 1,816–4,234 t).

**Table 2:** Negative binomial parameters for modeling Steller sea lion counts along the northwest coast of Washington State between December 2020–August 2021.

Season	n	Mean Steller sea lion counts	Negative binomial distribution size	Haulout correction factor
Winter	3	976.33	36.34	2.78
Spring	7	888.29	32.08	2.78
Summer	13	969.77	25.80	1.48

The n column designates the number of surveyed days used in calculating the mean. The haul out correction factors are derived from Olesiuk [49]. Abundance estimates are the result of a random draw from a negative binomial distribution, where the mean is the product of the seasonal mean count multiplied by the seasonal correction factor for the proportion of the Steller sea lion abundance hauled out and available to be counted (e.g., corrected winter abundance was 2,714). The seasons were defined as: winter (Dec-Jan), spring (Feb-Apr), and summer (May-Aug).

### Consumption of Chinook salmon

**Fig 5:**
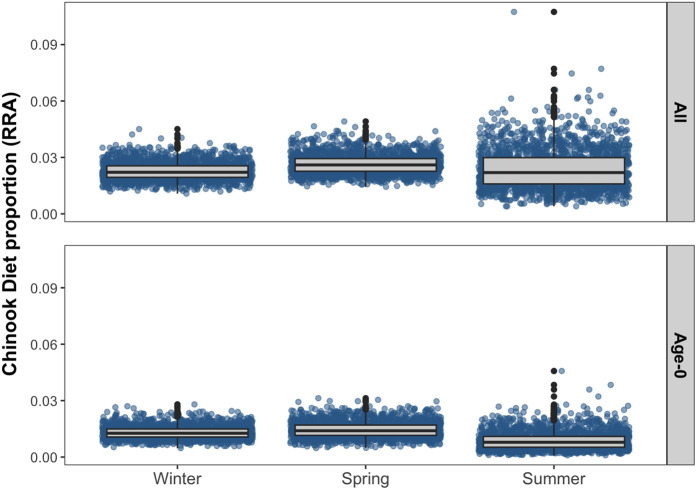
Zero/one inflated beta regression with Bayesian inference ‘zoib’ model derived diet proportions of Chinook salmon for 2,000 replicates per season. Box plots represent median (black line), 1^st^ and 3^rd^ quartiles of sample replicates.

**Fig 6:**
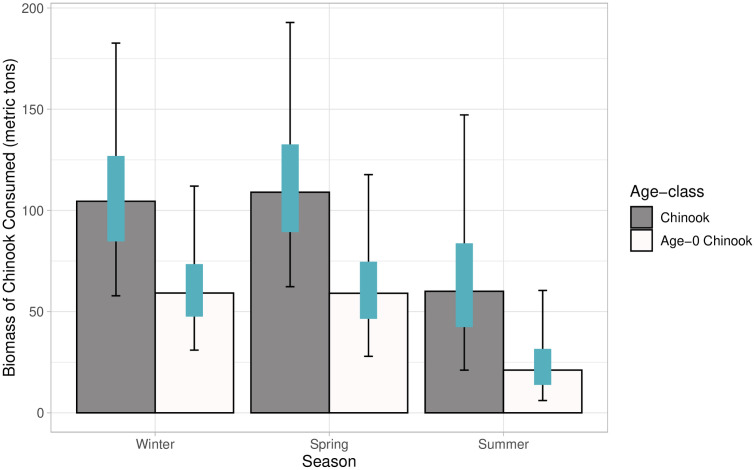
Biomass of Chinook salmon consumed by Steller sea lions along the northwest coast of Washington State from December 2020–August 2021. Metric tons of all (adult, unknown and age-0) Chinook salmon (black) and of age-0 Chinook salmon (grey) consumed. Bars represent model derived median, blue bars represent 50% predictive intervals, and black error bars represent 95% predictive intervals.

The estimated contributions of total Chinook salmon (all age classes) to Steller sea lion diet produced by the “zoib” models were relatively consistent across all seasons, with 95% CI of all three seasons overlapping (Fig 5). Pooled across all seasons, the estimated median proportion of Chinook salmon (all age classes) in Steller sea lion diet was 2.4% (95% CI: 1.1–4.1%). The proportion of Chinook salmon was marginally higher in spring (2.6%, 95% CI: 1.8–3.8%) compared to winter (2.2%, 95% CI: 1.5–3.3%) and summer (2.2%, 95% CI: 0.8–4.7%). Chinook salmon biomass consumed by Steller sea lions in northwest Washington varied seasonally over our study period (Fig 6). Total Chinook salmon biomass consumed was estimated at 284 t (95% PI: 191–417 t), with the highest biomass of Chinook consumed in the spring 109 t (95% PI: 62–193 t), followed by winter 105 t (95% PI: 58–183 t), and the lowest consumption occurring in the summer 60 t (95% PI: 21–147 t) (Fig 6).

### Age-0 consumption of Chinook salmon: models and sensitivity analysis

**Fig 7:**
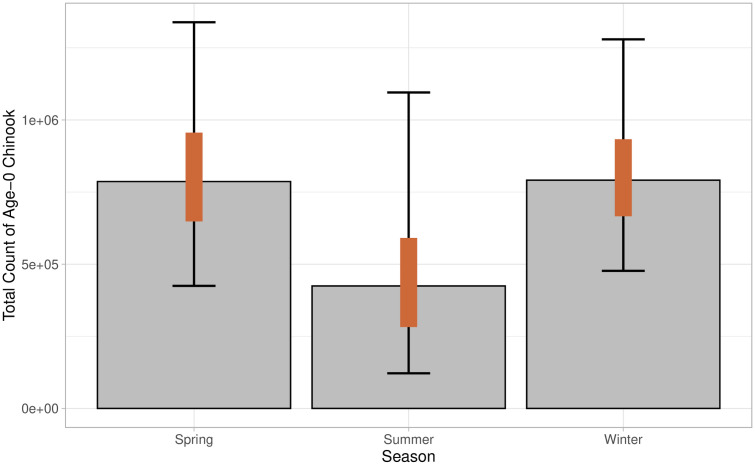
Seasonal count of age-0 Chinook salmon consumed by Steller sea lions along the northwest coast of Washington State from December 2020–August 2021. Bars represent model derived median, orange bars represent 50% predictive intervals, and black error bars represent 95% predictive intervals.

Age-0 Pacific salmon remains were used to determine the consumption of age-0 Chinook by Steller sea lions in our base model scenario (Table 1). The base model estimated the median proportion of age-0 Chinook salmon in scats was 1.2% (95% CI: 0.3–2.2%) across all seasons (Fig 5). The proportion of age-0 Chinook salmon were lowest in the summer (0.7%, 95% CI: 0.2–2.0), and similar in the spring (1.4%, 95% CI: 0.7–2.4), and winter (1.3%, 95% CI: 0.8–2.4). The biomass of ocean age-0 Chinook salmon consumed was estimated at 146 t (95% PI: 93–221 t) with 59 t (95% PI: 31–112 t) consumed in the winter, 59 t (95% PI: 28–118 t) consumed in the spring and 21 t (95% PI:6–60 t) in the summer (Fig 6). The base model estimated 2,064,418 (95% PI: 1,431,524–2,932,922) individual ocean age-0 Chinook were consumed during the study period (Fig 7). The highest estimated number of age-0 Chinook consumed was in winter (791,127 fish consumed, 95% PI: 476,946–1,279,366), followed by spring (786,408 fish, 95% PI: 424,955–1,338,671), and lowest in summer (424,573 fish consumed, 95% PI: 121,909–1,095,225).

**Fig 8:**
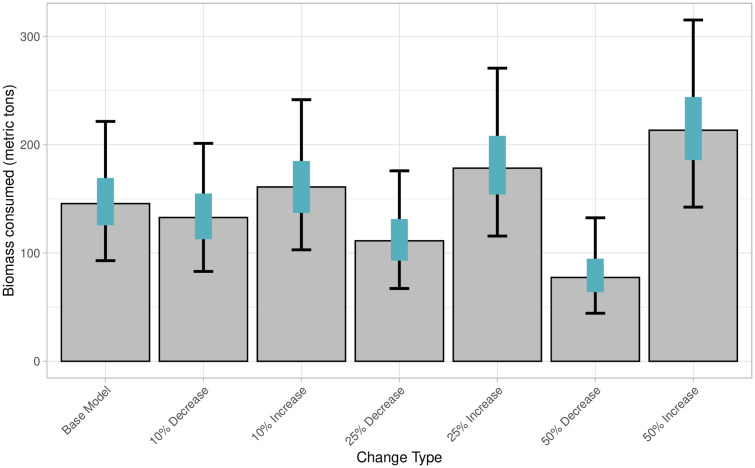
Total of biomass of age-0 Chinook salmon consumed by Steller sea lions along the northwest coast of Washington State from December 2020–August 2021 across base and alternate models for sensitivity analysis. Bars represent model derived median, blue bars represent 50% predictive intervals, and black error bars represent 95% predictive intervals.

**Fig 9:**
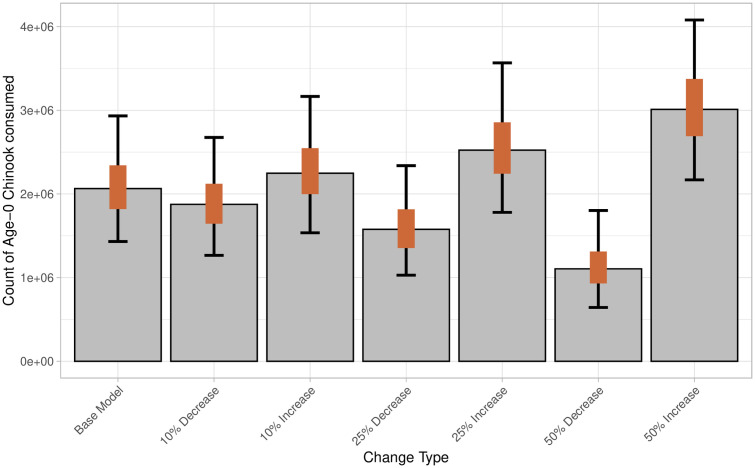
Total count of age-0 Chinook salmon consumed by Steller sea lions along the northwest coast of Washington State from December 2020–August 2021 across base and alternate models for sensitivity analysis. Bars represent model derived median, orange bars represent 50% predictive intervals, and black error bars represent 95% predictive intervals.

Six alternative models (increasing or decreasing age-0 proportion by 10%, 25%, and 50%) were generated to test the sensitivity of the modeling framework to the age-0 proportion inputs (Table 1). Across these models, the estimated median proportion of age-0 Chinook salmon in scats varied from 0.6% to 1.8% (S1 Fig). The median biomass of ocean age-0 Chinook salmon consumed varied from 77 to 213 t (Fig 8). The median count of individual ocean age-0 Chinook consumed varied from 1,105,141–3,010,663 across models (Fig 9). Results from the sensitivity test using bootstrapping methods showed that the age-0 biomass and count differed significantly from the base model in all alternative model cases (S2 and S3 Tables). Bootstrapping results showed that changes in age-0 proportion had overlapping 95% confidence intervals with the base model across all changes of age-0 proportions (Figs 8 and 9).

Estimated maximum individual daily consumption was highest in December and least in May for all Steller sea lion demographic groups in the base model (Table 3). Adult males had the highest estimated daily consumption of individual age-0 Chinook per day and juvenile females had the lowest estimate of age-0 Chinook consumed per day in all months of the base model; the total daily consumption of age-0 Chinook was similar for juvenile males and adult females.

**Table 3:** Median daily consumption of age-0 Chinook salmon by Steller sea lions along the northwest coast of Washington State between December 2020–August 2021.

Month	Juvenile Female	Juvenile Male	Adult Female	Adult Male
December	7.1 (3.7-13.3)	9.2 (4.6-17.7)	9.3 (5.4-16.6)	18 (8.9-33.7)
January	1.2 (0.6-2.2)	1.5 (0.8-2.9)	1.5 (0.9-2.6)	2.9 (1.5-5.6)
February	2.7 (1.4-4.9)	3.3 (1.7-6.3)	3.4 (1.9-5.8)	6.5 (3.2-12.4)
March	3.5 (1.7-6.8)	4.6 (2.1-9)	4.5 (2.3-8.3)	8.9 (3.9-17.5)
April	4.5 (2.1-8.8)	5.9 (2.7-11.4)	5.9 (3-10.8)	11.5 (5.2-22.5)
May	0.7 (0.3-1.4)	0.9 (0.4-1.8)	0.9 (0.5-1.7)	1.8 (0.8-3.6)
June	2.2 (0.6-6.4)	2.9 (0.8-8.2)	2.9 (0.9-8)	5.6 (1.5-16.5)
July	3.1 (0.9-9.1)	4 (1.1-12.4)	4.2 (1.2-11.3)	7.9 (2.3-22.9)
August	1.1 (0.3-3.1)	1.4 (0.4-4)	1.4 (0.4-4)	2.7 (0.8-7.9)

Median daily consumption was calculated using 2,000 bootstrapped Markov chain Monte Carlo simulations (MCMC) from each demographic group for each month and is presented as the number of fish consumed. The months with the maximum median consumption (December) and the minimum median consumption (May) are highlighted in grey. Numbers in parenthesis represent the 95% predictive intervals.

## Discussion

DNA metabarcoding diet analysis of Steller sea lions along the Washington coast showed a substantial increase in Chinook salmon detection and dietary contribution over the past decade. Chinook salmon were detected in 19% (FO) of Steller sea lion scats collected along the northwest coast of Washington during December 2020 through August 2021, compared to just 3% FO in scats from the same study region collected in 2010–2013 (excluding fall) that relied on genetic identification of Pacific salmon bones recovered from scat samples [72]. Further, 19% FO is markedly higher than the 0.7% FO reported in a meta-analysis of marine mammal diets spanning Southeast Alaska to central California over the past century [24]. This increased FO is likely driven by the higher taxonomic resolution, as well as increased number of prey items detected per scat with DNA metabarcoding [25,29,45,47,48]. The averaged estimated contribution of Chinook salmon to Steller sea lion diet during the present study was 2.4% (95% CI: 1.1–4.1%), with little variation between the winter, spring and summer periods. In contrast, during 2010–2013, Chinook salmon contributed only 1.1% (average of winter to summer) to the diet [31,55]. Thus, we estimate that the relative contribution of Chinook salmon to Steller sea lion diet is greater than was previously documented using other study methods. The observed increase in contribution of Chinook salmon to Steller sea lion diet can be partially attributed to the increased detection efficiency and species-level identification of salmon gained using DNA metabarcoding of the fecal material in the scat in comparison to earlier studies of Steller sea lion diet in the region [73–75].

The increased abundance of Steller sea lions during our study, as compared to 2010–2013, also resulted in a higher estimate of total prey biomass consumed. Seasonal haulout counts showed notable increases across all seasons: an 81% rise in winter, 37% in spring, and 30% in summer relative to the study conducted over 10 years ago [55]. These increases align with the observed increase in Steller sea lion abundance across the EDPS following their delisting [76]. The estimated total biomass of all prey items consumed by Steller sea lions in 2010–2013 was approximately 11,327 t per year [55]. Using the same modeling methods, prey biomass consumed from December 2020– August 2021 (9 months) was estimated at 11,802 metric tons (t; 95% CI: 9,228–15,005 t).

Increased consumption of Chinook by Steller sea lions over the past decade appear driven by these observed increases in sea lion abundance as well as the increased proportion of Chinook their diets. Our estimate of Chinook salmon consumption increased dramatically: from approximately 93.5 t per year in 2010–2013 [31] to 284 t (95% PI: 191–417 t) during just nine months of 2020–2021. Increases in the detection of Chinook salmon is in part due to improved detection efficiency with the use of DNA metabarcoding, but it is also likely that changes in ecological conditions influence our results. As generalist predators, an increase in consumption of Chinook salmon by Steller sea lions could be caused by increases in the availability of Chinook within the marine waters of northwest Washington or a reduction in the abundance or availability of other prey species, as noted in previous pinniped diet studies [55,77–79]. However, determining the total abundance of Chinook salmon along the coast of Washington is challenging, due to the mixing of stocks, spatial and temporal fluctuations in Chinook distribution, as well as increases in hatchery releases [13,14,34,42]. Therefore, it is difficult to corroborate the hypothesis that increased Chinook availability has directly led to higher diet proportions. Instead, it is more likely that a combination of updated modeling methods and changing environmental conditions has influenced Steller sea lion diets in the region [2,5,15,16,32,33].

### Implications of Steller sea lion predation to Chinook early marine survival

The present study supports the hypothesis that predation by individual Steller sea lions on age-0 Chinook salmon may play a larger role in low early marine survival than previously documented [4,5,24,27,28,31]. It is critical to note that age-0 Chinook consumption estimates must be interpreted with caution due to significant uncertainty, as indicated by large predictive intervals and the assumptions underlying model inputs. Despite this variability, findings from both the base and alternative models suggest an increase in Steller sea lions’ consumption of age-0 Chinook salmon from previous studies. Results from the base ‘zoib’ model, combined with aged salmon hard parts, estimated that age-0 Chinook salmon comprised 1.2% of the Steller sea lion diet—substantially higher than the 0.05–0.14% estimated by Chasco et al. [28] for the same region. The alternative model with the lowest proportion of age-0 Chinook consumed (50% reduction from the base model) estimated a diet proportion of 0.7% (S1 Fig), more than five times the previous diet proportions [28]. We estimate that Steller sea lions on the northwest coast of Washington consumed approximately 2 million age–0 Chinook salmon individuals between December 2020 and August 2021. Prior modeling studies estimated a significantly lower consumption across all marine mammal species (including pinnipeds and killer whales), with just over 0.7 million Chinook salmon (all age classes) consumed along the entire outer Washington coast in 2015 [28]. That same study estimated that across the entire north Pacific, Steller sea lions only consumed approximately 0.7 million Chinook (all ages) in 2015 [28]. Across all alternative models, the median estimate for Chinook salmon consumption ranged from 1.5 to 3 million fish (Fig 9) reinforcing the conclusion that the contribution of age-0 Chinook to the Steller sea lion diet is greater than earlier estimates suggested.

Evaluating the net impact of early marine mortality due to an individual predator species on adult Chinook salmon returns is complex, as mortality is likely neither exclusively compensatory nor exclusively additive [80]. Thus, assessing the impact of Steller sea lion predation based on biomass model results with high uncertainty is challenging to interpret in a broader ecological context. To provide a rough approximation of impact, we estimate that 2 million age-0 fish would equate to 14,771 adult (ocean age 2–4 years) Chinook salmon by multiplying maturity-at-age and survival-at-age data [28], assuming additive mortality. To put these numbers in a broader context, the total catch of ocean age 2 + Chinook salmon by state and tribal commercial ocean fishing fleets along the coast of Washington in 2022 was 60,343 fish [81]. This simple estimate suggests that predation mortality from Steller Sea lions during the study period and in the region is approximately 24% of the impact of commercial fishing fleets. It is important to note, however, that these landings do not include the number of fish caught in recreational fisheries and do not account for other fishery-related causes of mortality, such as due to hooking and release.

### Study assumptions and limitations

The use of DNA metabarcoding in this study may result in a variety of biases that ultimately affect prey sequence read proportions which in turn may introduce errors in the calculation of relative read abundance (RRA) for diet reconstruction and biomass quantification [45,60,82]. Errors in sample processing and data analysis include but are not limited to amplification bias [60], differences in bioinformatics [49,61,83], and errors in PCR amplification, i.e., “tag jumps” [84]. Correction factors based on both predator and prey species may refine RRA accuracy [29,51], however, feasibility is limited by the requirement of captive predator species and funding capacity [29]. In the absence of correction factors and to mitigate biases in both diet reconstruction methods, many diet reconstruction studies have been successful in cross validating RRA with alternative hard parts methods to corroborate results [25,45,47,52,85]. Deagle et al. [52] emphasized the importance of cross-validation across diet reconstruction methods, acknowledging the inherent biases in both hard remains and DNA-based approaches. Further, a review of previous pinniped diet studies by Thomas et al. [45] concluded that uncorrected RRA provides a higher taxonomic resolution of diet compared to hard parts frequency and yields diet proportions that are comparable to those obtained using other diet reconstruction methods, even without the application of correction factors. In this study, both uncorrected RRA and split-sample frequency (SSFO) from hard parts diet reconstruction methods produced nearly identical salmon diet estimates (6.5% vs. 6.7%; S3 Appendix). Overall, while uncorrected RRA is defensible for reconstructing Steller sea lion diets, the biases associated with DNA metabarcoding diet reconstruction methods should be considered when interpreting quantitative results.

The lack of Chinook salmon remains that could be aged posed a significant challenge in estimating the diet contribution of age-0 individuals. The primary method for determining salmon species and age in hard remains relies heavily on the recovery of otoliths [86,87], which are often poorly recovered from sea lion scats [86,88,89]. In this study, very few otoliths were recovered in sufficient condition for identification, and even fewer were identified to Chinook. The low recovery of Chinook otoliths in our scats may stem from various factors including their fragile nature [86,87], disproportionate degradation of the bones [90], regurgitation of large bones [91], or observed “belly biting” behavior in pinnipeds [86,92]. Further, biases in the recovery of certain ages of Chinook may result from size-selective foraging [93], differences in foraging behavior, depth, or location [94], or prey availability [56,79]. Due to the lack of species-specific hard remains, our model relied on remains identified at the genus level to determine the proportion of samples with age-0 fish. This presents a large potential source for bias in the age-0 consumption estimates, as this assumes that the Pacific salmon age-0 proportion is representative of the consumption of Chinook age-0 individuals.

To evaluate the impact of age assumptions on the biomass and number of individual age-0 Chinook salmon consumed, we conducted sensitivity tests with 10%, 25%, and 50% deviations from the base age-0 proportions. Bootstrapping showed significant changes in the biomass and number of individual Chinook consumed across all alternative models (S2 and S3 Tables). However, smaller changes (10% and 25%) had overlapping confidence intervals, suggesting similar distributions in the biomass consumed (Figs 8 and 9). Even with a 50% decrease in age-0 proportion (Table 1), the diet contribution of age-0 Chinook consumed increased compared to previous estimates. In contrast to our model, which assumes 51% of Chinook salmon consumed are from age-0 individuals, the Chasco et al. [28] model, based on preliminary data from Scordino et al. [46], assumed that 72% of all Chinook salmon consumed by Steller sea lions were age-0 fish (supplemental material, “Table_AverageSELAcrossMonths”). If we had assumed that 72% of Chinook salmon consumed were age-0, as in the Chasco paper [28], the results would more closely align with one of the alternative models (25% increase in age-0 proportion, Table 1), leading to a higher biomass and count of age-0 Chinook consumed (Figs 8 and 9).

Assumptions regarding the length of ocean age-0 Chinook in our models may introduce bias into the estimated total number of individual age-0 fish consumed by Steller sea lions. Our study lacked the resolution to reconstruct length of individual salmon consumed by the sea lions, and we used estimates of ocean age-0 lengths used previously for biomass modeling of Chinook along the coast of Washington [28]. Nelson et al. [30] explored differences in the size of salmon consumed by harbor seals as compared to the size distribution of salmon available in the environment and showed that the size of salmon consumed has a significant impact on the estimated number of individuals consumed. Thus, the total number of age-0 Chinook consumed during our study may be biased if the size distribution of age-0 Chinook in our study area differs from the size distributions previously estimated.

Finally, we assumed that Steller sea lion foraging on Chinook salmon did not vary between Tatoosh Island and Sea Lion Rock, or across all of the haulout sites in the study region. (Fig 1). It is possible that juvenile Chinook salmon are more accessible to Steller sea lions hauled out at the Tatoosh Island haulout complex due to the proximity of the site to Swiftsure Bank and the Juan de Fuca Eddy, which are historically known as aggregation sites for juvenile salmon [95]. However, we decided to expanded our study region based on three main lines of supporting evidence: 1) Steller sea lions along the northwest coast of Washington are multiple central place foragers [56] and utilize haulout sites throughout the region as past studies have found that sea lions branded for life-history studies [70] commonly use all haulout sites, including Tatoosh Island and Sea Lion Rock (Makah Fisheries Management unpublished data); 2) diet analysis from the same data used in this study [63] shows that Steller sea lions were generalist foragers of salmon; and 3) Steller sea lions at the three main haulout complexes in the region have a high degree of niche overlap based on both Shannon-Weiner and Morisita’s diversity indices for scat collections from 2010–2013 (Makah Fisheries Management unpublished data). The combined dietary studies along with branding studies suggest that Steller sea lions across the northwest coast of Washington show great mobility in foraging throughout the region and have access to and, are feeding on, similar prey items.

## Conclusions

In this study, we used metabarcoding data and applied modeling frameworks to estimate the biomass and number of Chinook consumed by Steller sea lions along the northwest coast of Washington during December 2020–August 2021. Our study marks the first biomass modeling framework incorporating Steller sea lion DNA metabarcoding data to match previous work produced for other pinniped species. Our consumption estimates of Chinook salmon by Steller sea lions were higher than estimates reported in previous studies of the region, which may be a factor of increased sea lion abundance, better data resolution, an increase in predation rates or some combination of those factors. We suggest that consistent monitoring across the Washington coast is necessary to better elucidate the impact of Steller sea lions on Chinook salmon. Further research should focus on producing more detailed data on Chinook salmon demographics and distribution along the coast of Washington to better understand potential impacts of Steller sea lion predation on specific Chinook salmon stocks and life stages. Our findings emphasize that updated modeling is necessary when considering the evolving predation impacts of growing Steller sea lion populations on Chinook salmon in this and other regions where the two species overlap.

## Supporting information

S1 Table Scat collection locations. Number of scats collected per season from Steller sea lions at Tatoosh Island and Sea Lion Rock along the northwest coast of Washington state.
(DOCX)

S1 Appendix ‘zoib’ modeling methods. (DOCX)

S2 Appendix Length and weight of Age-0 Chinook along the Washington coast. (DOCX)

S3 Appendix Split-sample frequency of occurrence calculations. (DOCX)

S1 Fig Bootstrapping sensitivity analysis results for the diet proportion of age-0 Chinook salmon consumed by Steller sea lions along the northwest coast of Washington State between December 2020-August 2021. Histograms of base and alternative models (n = 2,000 per model variation, per season) of the diet proportion of age-0 Chinook consumed by Steller sea lions for each season. Dashed lines represent median diet proportion for each model variation.
(TIF)

S2 Table Bootstrapping sensitivity analysis results for the median biomass consumed of age-0 Chinook salmon by Steller sea lions along the northwest coast of Washington State between December 2020-August-2021. Sample replicates did not demonstrate normal distribution; therefore, replicates were log transformed prior to bootstrapping and results are presented in terms of percent change from base model rather than observed difference.
(DOCX)

S3 Table Bootstrapping sensitivity analysis results for the median count of individual age-0 Chinook salmon consumed by Steller sea lions along the northwest coast of Washington State between December 2020-August-2021. Sample replicates did not demonstrate normal distribution; therefore, replicates were log transformed prior to bootstrapping and results are presented in terms of percent change from base model rather than observed difference.
(DOCX)
