# Supplementary material for: Steller sea lion (Eumetopias jubatus) consumption of ocean age-0 Chinook salmon (Oncorhynchus tshawytscha) along the northwest coast of Washington State
Source: PLoS One. 2025 Nov 12;20(11):e0334612. doi: 10.1371/journal.pone.0334612 (PMC12611116; doi:10.1371/journal.pone.0334612)
Supplement: S1 Table — Number of scats collected per season from Steller sea lions at Tatoosh Island and Sea Lion Rock along the northwest coast of Washington state. (DOCX) [file pone.0334612.s001.docx]

**S1 Table. Scat collection locations.** Number of scats collected per season from Steller sea lions at Tatoosh Island and Sea Lion Rock along the northwest coast of Washington state.

|  | **Winter** | **Spring** | **Summer** | **Total** |
| --- | --- | --- | --- | --- |
| **Tatoosh Island** | **94** | **60** | **61** | **215** |
| **Sea Lion Rock** |  | **30** | **29** | **59** |
