# Supplementary material for: Steller sea lion (Eumetopias jubatus) consumption of ocean age-0 Chinook salmon (Oncorhynchus tshawytscha) along the northwest coast of Washington State
Source: PLoS One. 2025 Nov 12;20(11):e0334612. doi: 10.1371/journal.pone.0334612 (PMC12611116; doi:10.1371/journal.pone.0334612)
Supplement: S1 Appendix — (DOCX) [file pone.0334612.s002.docx]

**S1 Appendix. ‘zoib’ modeling methods.**

Proportions of individual prey species in pinniped scats are non-negative continuous data with inflation at zero and one. Often, a very large fraction of scats samples contain zero salmon, and positive continuous values are right-skewed and heteroscedastic [1–3]. Thus, we assumed proportions of salmon in the Steller sea lion diet could be modeled with a beta distribution with inflation at 0 and/or 1, or Zero/One Inflated Beta Regression, hereafter as a “zoib” model [2,3]. The following description of the zoib model was adapted and condensed from Liu and Kong [4] and Liu and Eugenio [5], which provide more details on the zoib model formulation, including the full joint posterior distribution of the model, and descriptions of alternative priors.

Here, $y_{i}$ is a response variable (proportion of prey) measured on $n$ samples: $y_{i}=\left( y_{1},\ldots y_{n} \right)$. It was assumed that $y_{i}$ follows a piecewise distribution when $y_{i}$ has inflation at both 0 and 1, which is characteristic of the observed diet proportions of a given prey species in the sea lion scat samples:

(S2.1)
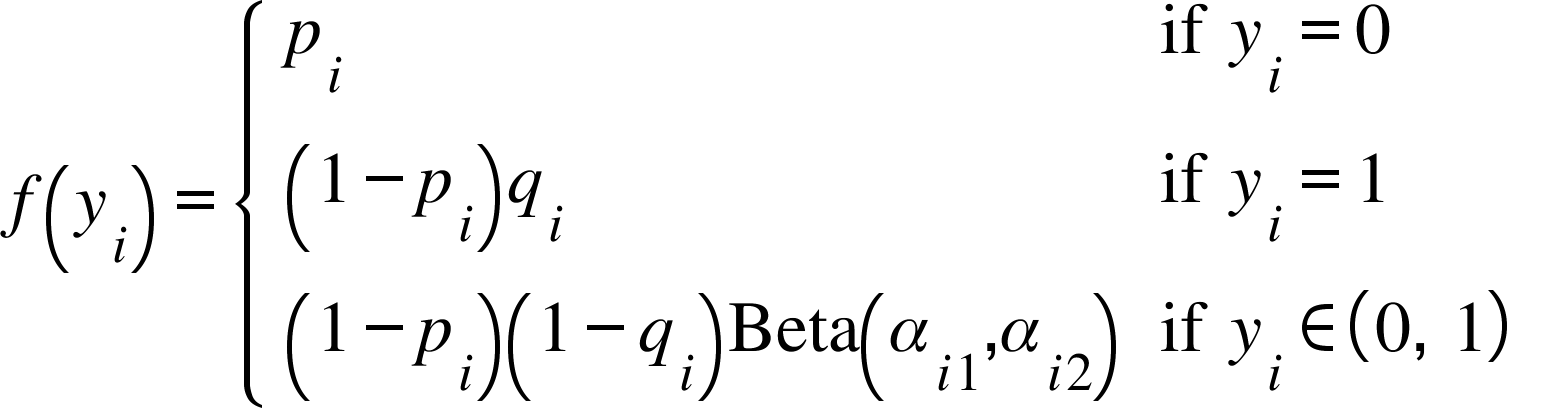


where $p_{i}$ is the probability that $y_{i}=0$, $q_{i}$ is the probability that $y_{i}=1|y_{i}\neq0$, and $\alpha_{i1}$ and $\alpha_{i2}$ are the two parameters of the beta distribution when $y_{i}\epsilon\left( 0, 1 \right)$. The parameters from the beta and binomial distributions are linked to the design matrix of explanatory variables ($\boldsymbol{x}_{\boldsymbol{i}}$**)** through the following link functions:

| (S2.2) | 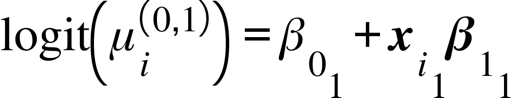 | (m = 1) |
| --- | --- | --- |
| (S2.3) | 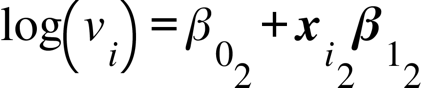 | (m = 2) |
| (S2.4) | 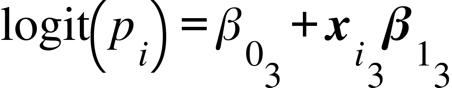 | (m=3) |
| (S2.5) | 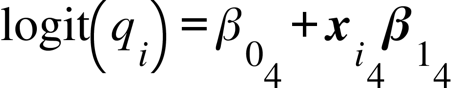 | (m = 4) |

where $\mu_{i}^{\left( 0,1 \right)}$ is the mean of the beta distribution $\mu_{i}^{\left( 0,1 \right)}=\alpha_{i1}\left( \alpha_{i1}+\alpha_{i2} \right)^{-1}$; $v_{i}$ is the sum of the two shape parameters of the beta distribution, $v_{i}=\alpha_{i1}+\alpha_{i2}$, also referred to as the “precision” parameter. The variance of the beta distribution can be calculated directly: $Var\left( y_{i}|y_{i}\in\left( 0,1 \right) \right)=\mu_{i}^{\left( 0,1 \right)}\left( 1-\mu_{i}^{\left( 0,1 \right)} \right)\left( v_{i}+1 \right)^{-1}. {\boldsymbol{\beta}_{1}}_{m}$are the fixed effects for each link function, $m$ ($m=1,2,3,4$). We imposed diffuse normal priors on the intercept and slope coefficients: ${\beta_{0}}_{m}\sim Normal\left( 0,{10}^{3} \right)$, ${\beta_{1}}_{m}\sim Normal\left( 0,{10}^{3} \right)$. We assumed the transformed mean and precision of the beta distribution (Eqs. 2 & 3) were functions of the sample collection season and were treated as fixed effects.

Model parameters were estimated with Bayesian inference using the ‘zoib’ package [4] in the R Programming Environment [6]. The ‘zoib’ package links the parameters in Eq. 1 to the explanatory variables in the link functions described above (Eqs. 2–5), then uses JAGS software [7] to implement Markov chain Monte Carlo simulations (MCMC). For both ‘zoib’ models, we generated four MCMC chains, each containing 4,000 samples, discarded the first half of each chain, then used the remaining 2,000 samples in each chain (8,000 total samples) to infer the marginal posterior distribution of each model parameter, and the posterior predictive distributions of each observation [8]. We assessed convergence of the MCMC chains by visually inspecting trace plots of all parameters, and calculation of the Gelman-Rubin diagnostic statistics [9]. Convergence of the MCMC chains was assumed if Gelman-Rubin statistics were less than 1.05, and there were no patterns in the trace plots that would suggest autocorrelation in the MCMC chains [8]. The distribution from these 8,000 samples were used to determine the 95% credible intervals (CI), and to create 2,000 sample replicates per season used in biomass modeling to determine both total and age-0 Chinook proportion of total biomass consumed.

**References**

1. Thomas AC, Nelson BW, Lance MM, Deagle BE, Trites AW. Harbour seals target juvenile salmon of conservation concern. Canadian Journal of Fisheries and Aquatic Sciences. 2017;74: 907–921. doi:10.1139/cjfas-2015-0558

2. Nelson BW, Pearson SF, Anderson JH, Jeffries SJ, Thomas AC, Walker WA, et al. Variation in predator diet and prey size affects perceived impacts to salmon species of high conservation concern. Canadian Journal of Fisheries and Aquatic Sciences. 2021;78: 1661–1676. doi:10.1139/cjfas-2020-0300

3. Liu L, Shih YCT, Strawderman RL, Zhang D, Johnson BA, Chai H. Statistical analysis of zero-inflated nonnegative continuous data: A review. Statistical Science. 2019;34: 253–279. doi:10.1214/18-STS681

4. Liu F, Kong Y. ZOIB: an R package for Bayesian inferences in beta and zero one inflated beta regression models. R J. 2015;7: 34–51.

5. Liu F, Evercita C Eugenio. A review and comparison of Bayesian and likelihood-based inferences in beta regression and zero-or-one-inflated beta regression. Stat Methods Med Res. 2018;27: 1024–1044. doi:0.1177/0962280216650699

6. R Core Team. 2017. R: A language and environment for statistical computing. R Foundation for Statistical Computing. Vienna, Austria. <https://www.R-Project.org/>.

7. Plummer, M. 2017. JAGS. <http://mcmc-jags.sourceforge.net/>.

8. Gelman, A., Carlin, J.B., Sterm, H.S., Dunson, D.B., Vehtari, A., Rubin, D.B. 2013. Bayesian data analysis. Third edition. CRC Press, Boca Raton, FL.

9. Gelman, A. and D. B. Rubin. 1992. Inference from iterative simulation using multiple sequences. Statistical Science 7:457-472.
