# Supplementary material for: Steller sea lion (Eumetopias jubatus) consumption of ocean age-0 Chinook salmon (Oncorhynchus tshawytscha) along the northwest coast of Washington State
Source: PLoS One. 2025 Nov 12;20(11):e0334612. doi: 10.1371/journal.pone.0334612 (PMC12611116; doi:10.1371/journal.pone.0334612)
Supplement: S2 Appendix — (DOCX) [file pone.0334612.s003.docx]

**S2 Appendix. Length and weight of Age-0 Chinook along the Washington coast**

The length distribution of age-0 Chinook salmon along the outer Washington coast is taken directly from modeling parameters in Chasco et al. [1]. The length distribution for age-0 Chinook salmon is an estimate based on monthly estimates of juvenile size, based on work by Teel et al. [2] and Weitkamp et al. [3]. Outer Washington Coast age-0 salmon sizes were based off the average size of hatchery released smolt lengths from the following release locations (as designated in RMIS) adjacent to the northwest Washington Coast study area (Grays harbor, Northern Washington Coast, Washington, general and Wilapa Bay). An additional 40 days of growth (to account for migration and residency times), at a rate of 1.0 mm/day [3] were used to estimate the monthly length estimates. Length estimates used for this region in Chasco et al. and in this model are shown in S2 Table 1. Preliminary data from a study of salmon along the coast of Washington state during the winter show that age-0 Chinook salmon (as determined by scale aging) had an average fork length of 26.9 cm (sd=3.7 cm, n=36; Makah unpublished data). These data are likely biased towards larger individuals, as this study is not targeting age-0 fish, and larger gear may be providing size selectivity towards larger individuals. Regardless, we acknowledge that estimates of age-0 Chinook length in this region are coarse, however, Chinook salmon at this life stage in this region remain understudied. The use of these estimates further highlights the importance of increased investigation of early marine stage Chinook to better understand both this life stage and mortality.

To account for variability in age-0 Chinook length, monthly average fork lengths were modeled using a log normal distribution with a standard deviation of 0.5, truncated at a minimum length of 100 mm and a maximum length of 300 mm (the maximum size for age-0 Chinook salmon designation in this paper [4,5]) with 2,000 replicates per month (Fig 1; S2). The minimum length is an estimate based on data from the Puget Sound that shows the release-size of hatchery fish is typically around 100 mm [6,7]. Although these data only pertain to hatchery fish, which are usually larger than natural origin fish, choosing a higher minimum bound provides a conservative estimate (i.e. less Chinook individuals consumed per kg). These monthly fork lengths were then converted into weight (in kg) by the allometric model reported in Nelson et al. [4], to get sample replicates of age-0 Chinook sample weights.

S3 Table: Average monthly length of age-0 salmon from Chasco et al. [1] in millimeters.

| December | January | February | March | April | May | June | July | August |
| --- | --- | --- | --- | --- | --- | --- | --- | --- |
| 237.00 | 175.44 | 170.27 | 202.00 | 234.86 | 173.67 | 142.02 | 133.11 | 151.50 |

**S3 Fig A: Log-normal distribution of age-0 Chinook salmon lengths, where mean length is represented by dashed red lines.**


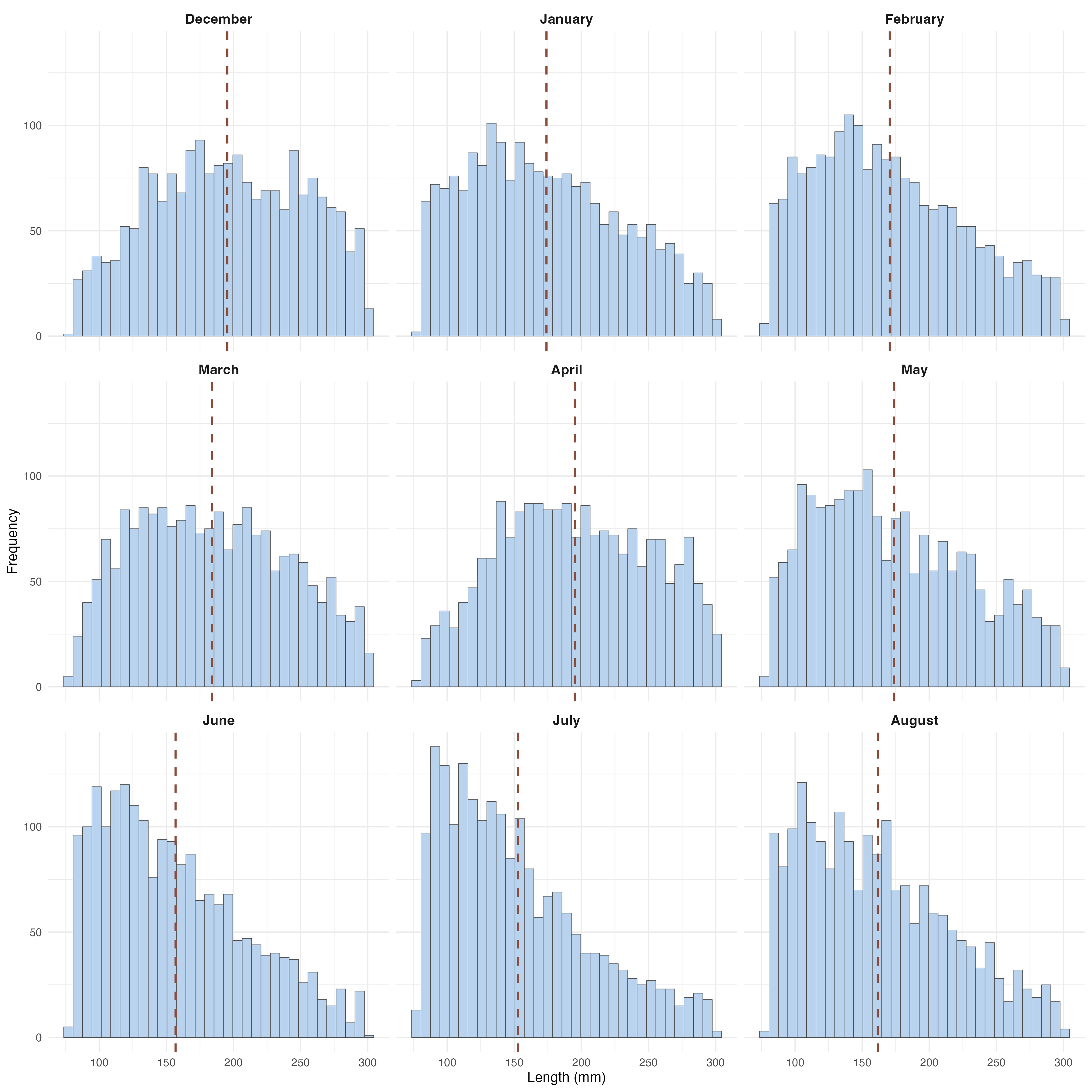


**S3 Fig B: Distribution of age-0 Chinook salmon weights, where mean length is represented by dashed red lines.**


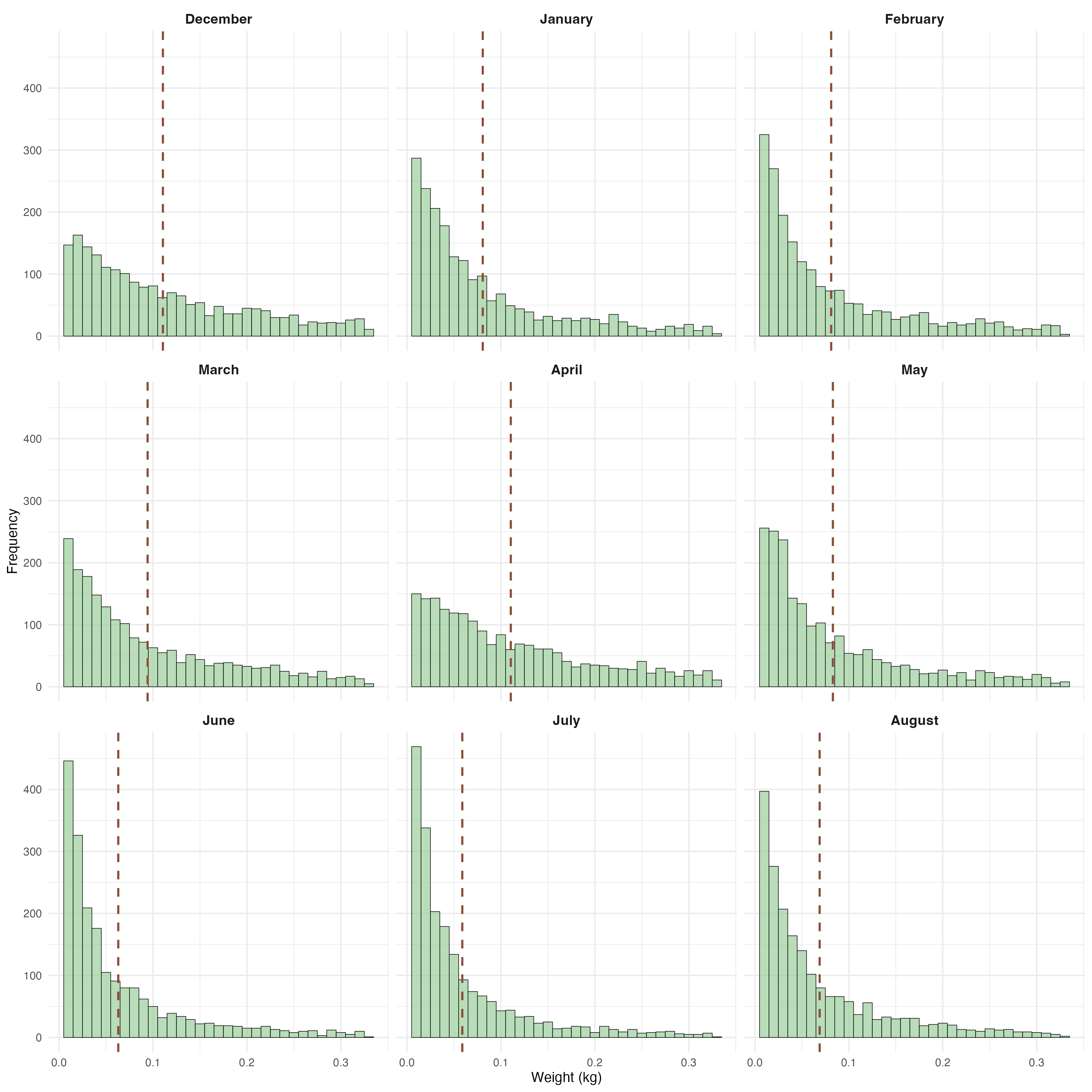


**References**

1. Chasco B, Kaplan IC, Thomas AC, Acevedo-Gutiérrez A, Noren DP, Ford MJ, et al. Competing tradeoffs between increasing marine mammal predation and fisheries harvest of Chinook salmon. Sci Rep. 2017;7: 1–14. doi:10.1038/s41598-017-14984-8

2. Teel DJ, Burke BJ, Kuligowski DR, Morgan CA, Van Doornik DM. Genetic Identification of Chinook Salmon: Stock-Specific Distributions of Juveniles along the Washington and Oregon Coasts. Marine and Coastal Fisheries. 2015;7: 274–300. doi:10.1080/19425120.2015.1045961

3. Weitkamp LA, Teel DJ, Liermann M, Hinton SA, Van Doornik DM, Bentley PJ. Stock-specific size and timing at ocean entry of columbia river juvenile chinook salmon and steelhead: Implications for early ocean growth. Marine and Coastal Fisheries. 2015;7: 370–392. doi:10.1080/19425120.2015.1047476

4. Nelson BW, Pearson SF, Anderson JH, Jeffries SJ, Thomas AC, Walker WA, et al. Variation in predator diet and prey size affects perceived impacts to salmon species of high conservation concern. Canadian Journal of Fisheries and Aquatic Sciences. 2021;78: 1661–1676. doi:10.1139/cjfas-2020-0300

5. MacLellan SE, Gillespie DC. Chinook Salmon (*Oncorhynchus tshawytscha*) Scale Age Determination Procedures. Can Tech Rep Fish Aquat Sci. 2015.

6. Nelson BW, Shelton AO, Anderson JH, Ford MJ, Ward EJ. Ecological implications of changing hatchery practices for Chinook salmon in the Salish Sea. Ecosphere. 2019;10. doi:10.1002/ecs2.2922

7. Gamble MM, Connelly KA, Gardner JR, Chamberlin JW, Warheit KI, Beauchamp DA. Size, Growth, and Size-Selective Mortality of Subyearling Chinook Salmon during Early Marine Residence in Puget Sound. Trans Am Fish Soc. 2018;147: 370–389. doi:10.1002/TAFS.10032
