# Supplementary material for: Steller sea lion (Eumetopias jubatus) consumption of ocean age-0 Chinook salmon (Oncorhynchus tshawytscha) along the northwest coast of Washington State
Source: PLoS One. 2025 Nov 12;20(11):e0334612. doi: 10.1371/journal.pone.0334612 (PMC12611116; doi:10.1371/journal.pone.0334612)
Supplement: S3 Appendix — (DOCX) [file pone.0334612.s004.docx]

**S3 Appendix. Split-sample frequency of occurrence calculations.**

Split-sample frequency of occurrence (SSFO) compares the relative proportion of a given prey item within each sample across a population of samples [1]. The result of the SSFO calculation is a percentage, where the presence/absence of a prey item in each scat sample is averaged across all scats for each prey type. In other words, if there are 10 prey taxa present in one sample, each prey taxa has a resulting 10% SSFO, using the calculation presented in the following equation.

Equation 1: Split-sample frequency of occurrence ﻿(SSFO_i_)

$$SSFO_{i}= \frac{\sum_{k=1}^{i} {O_{ik}}/{O_{k}}}{s}$$

where;

O_ik_ = 0 if taxon i is absent in fecal k

= 1 if taxon i is present in fecal k

O_k_ = ﻿total number of all taxa present in fecal k

s = total number of fecal samples that contained prey

SSFO may be used as a proxy for diet composition, and is used in this study with both prey items detected in hard remains as well as prey items detected used DNA metabarcoding. Prey presence via hard parts analysis is determined by species or family level identification of hard parts (e.g. fish bones, cartilaginous structures, and cephalopod pens and beaks) found within an individual scat sample. Prey presence via DNA metabarcoding analysis was determined if prey taxa reads composed >1% of the total prey reads within a sample.

**S4 Table**: A comparison of diet proportion metrics used to estimate diet of Steller sea lions along the coast of northwest Washington State between December 2020–August 2021. Relative read abundance (RRA) from DNA metabarcoding and split-sample frequency of occurrence (SSFO) from both hard parts and SSFO from DNA metabarcoding were calculated for the proportion of Salmonidae. Species level calculations were performed for both DNA based metrics, but hard part data was excluded due to low recovery of hart parts with species level identification of salmon.

|  |  |  |  |
| --- | --- | --- | --- |
|  | RRA from DNA | SSFO hard part | SSFO from DNA |
| Salmon Family | 6.7% | 6.2% | 22.6% |
| Chinook Salmon | 2.3% | – | 3.8% |
| Coho Salmon | 2.2% | – | 5.0% |
| Pink Salmon | 1.4% | – | 4.5% |
| Steelhead | 0.7% | – | 1.4% |
| Sockeye Salmon | <0.1% | – | 1.2% |
| Chum Salmon | <0.1% | – | 0.4% |
| Brown Trout | <0.1% | – | <0.1% |

**References**

1. Olesiuk PF, Bigg MA, Ellis GM, Crockford SJ, Wigen RJ. An Assessment of the Feeding Habits of Harbour Seals (*Phoca vitulina*) in the Stait of Georgia, British Columbia, Based on Scat Analysis. Can Tech Rep Fish Aquat Sci. Nanaimo, British Columbia; 1990.
