# Supplementary figures and images for: Steller sea lion (Eumetopias jubatus) consumption of ocean age-0 Chinook salmon (Oncorhynchus tshawytscha) along the northwest coast of Washington State
Source: PLoS One. 2025 Nov 12;20(11):e0334612. doi: 10.1371/journal.pone.0334612 (PMC12611116; doi:10.1371/journal.pone.0334612)

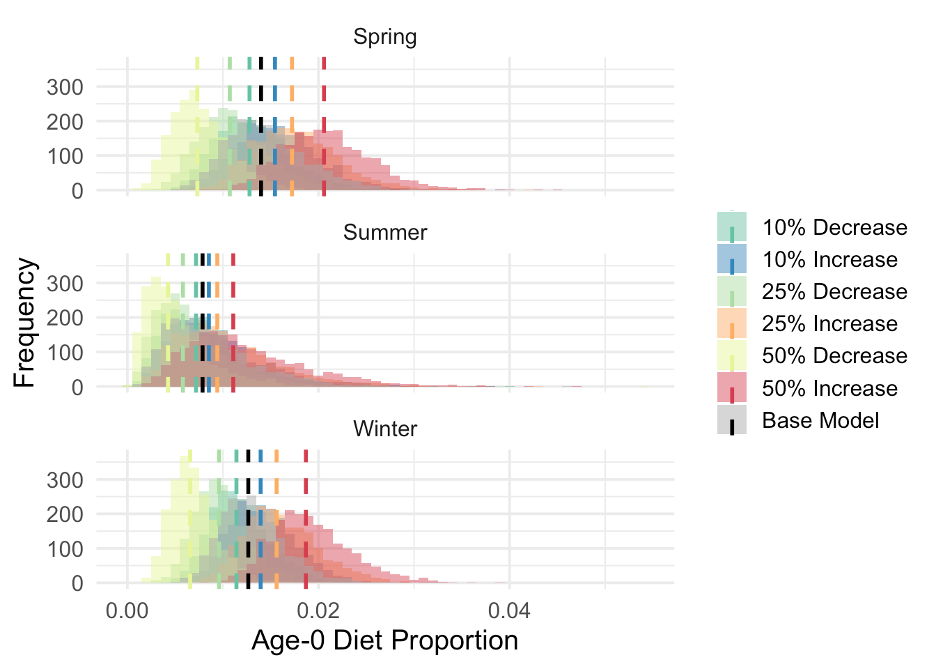

Supplement: S1 Fig — Histograms of base and alternative models (n = 2,000 per model variation, per season) of the diet proportion of age-0 Chinook consumed by Steller sea lions for each season. Dashed lines represent median diet proportion for each model variation. (TIF) [file pone.0334612.s005.tif]
