# Supplementary material for: Steller sea lion (Eumetopias jubatus) consumption of ocean age-0 Chinook salmon (Oncorhynchus tshawytscha) along the northwest coast of Washington State
Source: PLoS One. 2025 Nov 12;20(11):e0334612. doi: 10.1371/journal.pone.0334612 (PMC12611116; doi:10.1371/journal.pone.0334612)
Supplement: S2 Table — Sample replicates did not demonstrate normal distribution; therefore, replicates were log transformed prior to bootstrapping and results are presented in terms of percent change from base model rather than observed difference. (DOCX) [file pone.0334612.s006.docx]

**S2 Table. Bootstrapping sensitivity analysis results for the median biomass consumed of age-0 Chinook salmon by Steller sea lions along the northwest coast of Washington State between December 2020-August-2021.** Sample replicates did not demonstrate normal distribution; therefore, replicates were log transformed prior to bootstrapping and results are presented in terms of percent change from base model rather than observed difference.

|  | **Median biomass consumed (with 95% PI)** | **Inverse log of observed difference** | **P Value** | **Change from base model** |
| --- | --- | --- | --- | --- |
| Base Model | 145.6 (92.9-221.5) |  |  |  |
| 10% Increase | 161 (102.9-241.5) | 1.0918 | p <0.0001 | 9.18 % |
| 10% Decrease | 132.8 (83-201.2) | 0.9061 | p <0.0001 | -9.39 % |
| 25% Increase | 178.3 (115.6-270.6) | 1.2279 | p <0.0001 | 22.79% |
| 25% Decrease | 111.2 (67.2-175.9) | 0.7602 | p <0.0001 | -23.97% |
| 50% Increase | 213.3 (142.3-315.1) | 1.4614 | p <0.0001 | 46.14% |
| 50% Decrease | 77.4 (44.4-132.5) | 0.5317 | p <0.0001 | -46.83% |
