# Supplementary material for: Steller sea lion (Eumetopias jubatus) consumption of ocean age-0 Chinook salmon (Oncorhynchus tshawytscha) along the northwest coast of Washington State
Source: PLoS One. 2025 Nov 12;20(11):e0334612. doi: 10.1371/journal.pone.0334612 (PMC12611116; doi:10.1371/journal.pone.0334612)
Supplement: S3 Table — Sample replicates did not demonstrate normal distribution; therefore, replicates were log transformed prior to bootstrapping and results are presented in terms of percent change from base model rather than observed difference. (DOCX) [file pone.0334612.s007.docx]

**S3 Table. Bootstrapping sensitivity analysis results for the median count of individual age-0 Chinook salmon consumed by Steller sea lions along the northwest coast of Washington State between December 2020-August-2021.** Sample replicates did not demonstrate normal distribution; therefore, replicates were log transformed prior to bootstrapping and results are presented in terms of percent change from base model rather than observed difference.

|  | **Median** | **Inverse log of observed difference** | **P value** | **Percent change from base model** |
| --- | --- | --- | --- | --- |
| Base Model | 2,064,418  (1,431,524-2,932,922) |  |  |  |
| 10% Increase | 2,247,983  (1,535,813-3,165,494) | 1.0902 | p <0.0001 | 9.01 % |
| 10% Decrease | 1,875,745  (1,266,216-2,675,260) | 0.9056 | p <0.0001 | -9.44 % |
| 25% Increase | 2,523,218  (1,780,010-3,566,843) | 1.2248 | p <0.0001 | 22.48 % |
| 25% Decrease | 1,577,026  (1,029,159-2,337,896) | 0.7599 | p <0.0001 | -24.01 % |
| 50% Increase | 3,010,663  (2,168,187-4,079,243) | 1.4567 | p <0.0001 | 45.67 % |
| 50% Decrease | 1,105,141  (643,241-1,802,240) | 0.5342 | p <0.0001 | -46.58 % |
